# Virtual Reality (VR) in Assessment and Treatment of Addictive Disorders: A Systematic Review

**DOI:** 10.3389/fnins.2019.01409

**Published:** 2020-01-10

**Authors:** Tomoyuki Segawa, Thomas Baudry, Alexis Bourla, Jean-Victor Blanc, Charles-Siegfried Peretti, Stephane Mouchabac, Florian Ferreri

**Affiliations:** ^1^Department of Psychiatry, Hôpital Saint-Antoine, Sorbonne Université, Paris, France; ^2^Jeanne d'Arc Hospital, INICEA Group, Saint-Mandé, France

**Keywords:** virtual reality, addictive behavior, alcohol, nicotine, cocain

## Abstract

**Background:** Substance Use Disorder (SUD) and behavioral addictions are common and require a multidisciplinary approach. New technologies like Virtual Reality could have the potential to improve assessment and treatment of these disorders.

**Objective:** In the present paper, we therefore present an overview of Virtual Reality (Head Mounted Devices) in the field of addiction medicine for craving assessment and treatment.

**Method:** We conducted a systematic review by querying PubMed database for the titles of articles published up to March 2019 with the terms [virtual] AND [addictive] OR [addiction] OR [substance] OR [alcohol] OR [cocaine] OR [cannabis] OR [opioid] OR [tobacco] OR [nicotine] OR [methamphetamine] OR [gaming] OR [gambling].

**Results:** We screened 319 abstracts and analyzed 37 articles, dividing them into two categories, the first for assessment of cue reactivity (craving, psychophysiological response and attention to cue) and the second for intervention, each drug (nicotine, cocaine, alcohol, cannabis, gambling) being detailed within each category.

**Conclusions:** This overview suggest that VR provide benefits in the assessment and treatment of substance use disorders and behavior addictions and achieve high levels of ecological validity. While, craving provocation in VR is effective across addiction disorders, treatments based exclusively on virtual exposure to drug related cues as shown heterogenous results.

## Introduction

Substance Use Disorder (SUD) and behavioral addictions are prevalent in many countries (Degenhardt et al., 2017) and require a multidisciplinary approach. Unfortunately, only a minority (7.1%) of patients, even in high income countries, receives adequate treatment. Individuals diagnosed with SUD experience several relapses after interventions and a lower quality of life because of the chronic nature of these disorders (Degenhardt et al., 2017). Therefore, there is an urgent need to conduct more research to expand assessment and treatment approaches. Virtual reality (VR) is emerging as one of the technological keys, it is increasingly accessible, and easy-to-use, and has recently attracted attention because of its potential utility for individuals with SUD (Carter and Tiffany, 1999).

VR is a computer-generated simulation that is a set of images and sounds that represents a real place or situation, which can be interacted with, in a seemingly real or physical way by a person using special electronic equipment. It can transmit visual, auditory, and various sensations to users through a headset to make them feel as if they are in a virtual or imagined environment.

Cue reactivity, which is based on classical and instrumental conditioning theory, involves conditioned response such as craving, psychophysiological response (heart rate, skin conductance…), attention bias and drug seeking behaviors triggered by stimuli previously associated with drug use (Ferreri et al., 2018). Traditional cue reactivity studies (scripts, photographs, videos and objects related to drug use), have allowed for a better understanding of situations leading to continued use and factors triggering relapse (Conklin and Tiffany, 2002). However, many limitations exist: lack of standardization, control and generalization, lack of contextual, and complex cues variety resulting in limited ecological validity and decreasing extinction probability through cue-exposure therapy. Therefore, because VR is immersive, both the environment and the perceptual stimuli can be modulated to trigger and assess pathological behaviors or sensations (e.g., craving), as well as to evaluate behavioral responses to a given situation that can elicit distress. Patients cans learn how to cope with their problems better. Beyond the use for addictive psychopathologies it has been shown to be useful for other psychiatric disorders by Park et al. (2019).

Moreover, VR has the potential to ameliorate many issues like craving assessment; active treatment of craving using cue exposure therapy (CET) or cognitive behavioral therapy (CBT)-driven techniques applied in real time. VR may also facilitate the link between clinician and patients and improve our understanding of addictive behavior.

Until recently, VR was limited by its cost and by the quality of the multimedia content. There has been a recent democratization of these systems (Playsatation4 VR, Oculus Rift, etc.) concomitant with the video game industry's growing interest in this technology. Decreasing costs and increasing power are making it useful for performing an ecological assessment of cognition, emotions, and behavior in real-time.

The purpose of this systematic review is to evaluate the usefulness and efficacy of immersive VR in cue reactivity assessment and craving management for patients undergoing SUD or behavioral addictions. To this end, the proposed systematic review will answer the following questions:

- When compared to VR neutral stimuli, are VR cues able to elicit cue reactivity in adult patients suffering from addictive disorders? Is there an advantage of using VR cue reactivity over traditional cue reactivity methods?
- Can VR be used as an effective tool for craving reduction compared to standard therapy in this aforementioned population?

## Method

### Eligibility Criteria

To identify appropriate resources and search for relevant evidence. We used a PICOS framework, to form a well-focused question and facilitate the literature search (Schardt et al., 2007).

**Table:** 

Population	Adolescent or adult humans with SUD or behavioral addiction
Intervention	Immersive VR (using Head-Mounted Display) simulating drug-related cues for assessment or treatment
Comparators	- Assessment: virtual neutral stimuli por traditional exposition (photos, videos, imagination), *in vivo* - Treatment: traditional therapy (CET, CBT, replacement therapy)
Outcomes	- Assessment: variation of cue reactivity (level of craving, physiological responses, attention to cue) - Treatment: level of craving, level of dependence, maintenance of abstinence
Study designs	Randomized controlled trial (RCT), controlled trial (CT), trial (T), case series
Timing	Studies running up to March 2019
Language	English or French

We conduct a systematic review by querying PubMed and Embase using the MeSH terms and keywords [virtual] AND [addictive] OR [addiction] OR [substance] OR [alcohol] OR [cocaine] OR [cannabis] OR [opioid] OR [tobacco] OR [nicotine] OR [methamphetamine] OR [gaming] OR [gambling]. TS and TB screened 471 articles, and 37 were then analyzed and divided into two categories, the first for assessment of cue reactivity (craving, psychophysiological response and attention to cue) and the second for treatment, each drug being detailed within each category.

**Figure 1:**
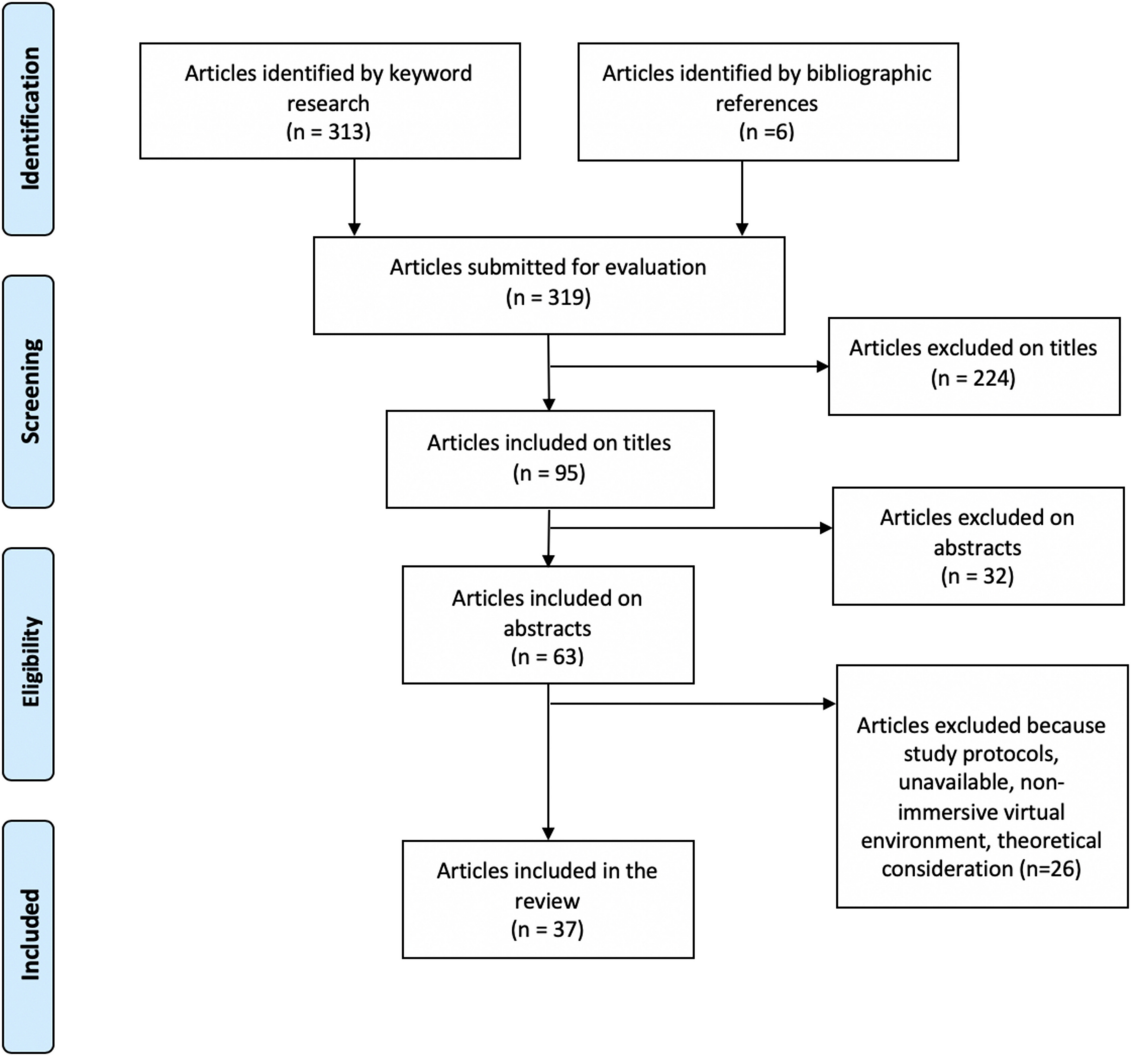
PRISMA flow diagram.

See PRISMA diagram (Figure 1).

## Results

### Conceptual Overview

A brief description of the concepts underlying virtual reality (VR) and cue features in the field of addiction is summarized in Table 1.

**Table 1:** Summary of concepts underlying Virtual Reality and type of cue.

**Concept**	**Description**	**References**
Virtual reality	An advanced human-computer interface that simulates a realistic environment and allows participants to interact with it. Its purpose is to allow a person sensory-motor and cognitive activity in an artificial world, created numerically, which can be imaginary, symbolic or a simulation of certain aspects of the real world. Each VR application is characterized by two key criteria: presence and autonomy.	Ouramdane et al., 2009
Virtual environment	The place suggested by the VR, represented by a 3D model of real or imaginary data that can be visualized and with which participants can interact in real time.	Ouramdane et al., 2009
Immersion	The objective description of what a VR display can provide in terms of technologies. It includes the extent to which the display is extensive (number of sensory systems involved), surrounding (information can arrive from any direction), inclusive (all information from the real world is shut out), vivid (richness and quality of the sensory information generated) and matching (a match is needed between information generated and participant's proprioceptive feedback). It also requires a self-presentation in the virtual environment i.e., a virtual body.	Slater et al., 1999
Presence	The subjective, psychological feeling of “being there”, in the place depicted by the virtual environment. Immersion, control over environments, naturalness and realness of interactions all together contribute to the sense of presence.	Witmer and Singer, 1998
Head-Mounted Display (HMD)	The most immersive device: it displays separated images for each eye allowing stereo vision with stereo earphones and head tracking continually capturing the position and orientation of the participant's head. Rather than being a passive, external observer of video images, it allows participants to see a surrounding 3D stereo scene that can change dynamically.	Anderson et al., 2001; Slater, 2009
Cybersickness	A constellation of motion sickness-like symptoms occurring during and upon VR exposure. It includes symptoms such as disorientation, dizziness and nausea. and may be considered as a potential threat to the ultimate usability of virtual reality.	Stanney et al., 1997
Craving	Defined by the subjective preoccupation or strong desire to use a drug, craving has become a major diagnostic criterion of addictive disorders and is considered a central feature of addiction.	Sayette, 2016
Psycho-physiological response	These responses, controlled by the autonomic nervous system, are considered objective markers of cue-reactivity. Heart rate, skin conductance and temperature are the most studied psychological responses.	Conklin and Tiffany, 2002
Attention to cue	Referring to attention bias, attention to cue is the motivational trend to focus on drug cues while neglecting or ignoring others type of stimuli.	Field et al., 2014
Proximal cues	This is the most frequent type of cue used in traditional cue-reactivity studies. Proximal cues are ubiquitous across drug use. They are more often visual cues such as cigarette, ashtray, lighters, bottle of alcohol but can also be olfactory, auditory and tactile.	Conklin et al., 2008
Contextual (or distal) cues	They refer to the environment or context, with or without social interaction, in which substance use occurs such as bar or party. As well as proximal cues and despite being less reliable, they can elicit conditioned responses by being previously paired with drug use.	Conklin et al., 2008
Complex cues	A combination of proximal and contextual cues. They represent a more complete picture of real-world stimuli (people drinking alcohol in a party or smokers gathering outside a bar).	Conklin et al., 2008

### Assessment

Studies and results described below are summarized in Table 2.

**Table 2:** Clinical trial on Virtual Reality (VR) assessment of craving in addiction.

**Study**	**Population**	**VR procedure**	**Assessment**	**Outcome**
**NICOTINE**				
Lee et al. (2003)	−22 TS, moderate ND^a^	- C: complex/CC: smoking-related pictures - Randomized, 5 min exposure	- VAS	- ↑ Craving
Lee et al. (2005)	−8 TS, moderate ND^a^	- C: complex/CC: smoking-related pictures or VR neutral - Randomized, 5 min exposure	- VAS - fMRI	- ↑ CravingActivation of PFC
Bordnick et al. (2004)	−13 NTS, ND	- C: proximal or complex cues/CC: VR neutral - Randomized, 3 min exposure each	- VAS	- ↑ Craving
Bordnick et al. (2005)	−10 NTS, ND		- VAS - SCR	- ↑ Craving and SCR
Carter et al. (2008)	−22 NTS, ND^b^	- C: complex/CC: VR neutral - 3 min exposure	- MDS, brief QSU items	- ↑ Craving
Traylor et al. (2008)	−20 NTS, moderate ND^a^, ^b^	- C: proximal or complex cues/CC: VR neutral - Randomized, 3 min exposure	- VAS,	- ↑ Craving
Traylor et al. (2009)	−20 NTS, moderate ND^a^, ^b^	- C: proximal or complex/CC: VR neutral - Randomized, 3 min exposure	- ACVAS, SoSQ	- ↑ Attention to cues - ↑ Attention to sight in proximal vs. complex environment
Ferrer-García et al. (2010)	−25 NTS, smokers (>10 cig/d)	- C: 7 complex/CC: VR neutral - Randomized, 6 min exposure to each scene	- VAS, cig/day - PQ	- ↑ Craving - Presence significantly correlated with craving
Traylor et al. (2011)	−14 NTS, moderate ND^a^, ^b^/intermediate AD^b^, ^c^ - Control: 7 NTS, moderate ND^a^, ^b^/daily drinkers	- C: 2 complex/CC: VR neutral - 3 min exposure	- VAS	- ↑ Craving
Pericot-Valverde et al. (2011)	−46 NTS, ND^b^	- C: 2 complex (+/– social pressure) - Randomized, 6 min exposure	- VAS	- ↑ Craving
Kaganoff et al. (2012)	−46 TS (NRT +/– CBT), ND^b^	- C: proximal or complex/CC: VR neutral - Randomized exposure	- Intake, week 4 and week 10 of treatment: VAS, SAS	- ↑ Craving - ↑ Attention to cue
Paris et al. (2011)	−24 NTS, smokers (>10 cig/day)	- C: complex or contextual/CC: 2 VR neutral - 3 min exposure	- VAS	- ↑ Craving - ↑ Craving in complex vs. contextual environment - ↑ Craving between last and first neutral environment
García-Rodríguez et al. (2012)	−46 NTS, smokers (>10 cig/day) - 44, no smokers	- C: 7 complex/CC: VR neutral - Randomized, 6 min exposure	- VAS - HR, SR, T	- ↑ Craving in smoker group. - ↓ HR: in five environments in smoker group - ↑ T in two environments in smoker group - No difference in SR
García-Rodríguez et al. (2013)	−45 NTS, smokers (>10 cig/day)	- C: Smoking a virtual cigarette in complex environment/CC: virtual darts in complex environment or complex environment alone - Randomized, 6 min exposure	- VAS - HR	- ↑ Craving - ↑ HR
Acker and MacKillop (2013)	−47 NTS, low to moderate ND^1^ (>10 cig/day)	- C: proximal/CC: VR neutral - 3 min exposure	- VAS - Cigarette Purchase Task and self-administration paradigm	- ↑ Craving - ↑ Motivational value - Craving and motivational indices correlated to number of cigarettes purchased
Gamito et al. (2014)	−21 NTS, low ND^a^ - 24, no smokers	- C: complex / CC: contextual - 5 min exposure	- QSU-Brief - Eye tracking in complex cue exposure - PQ, SSQ	- ↑ In smoker group in complex environment - ↑ Eye fixations on cues in smoker group
Thompson-Lake et al. (2015)	−36 NTS, high ND^a^, ^b^ (one overnight nicotine deprived)	- C: proximal or complex. CC: VR neutral—Randomized, 3 min exposure	- QSU-Brief, FTND, Withdrawal Scale (12 h Deprived) - ACVAS - HR	- ↑ Craving - ↑ Craving in complex vs. proximal environment - Craving correlated with dependence and withdrawal in complex environment - ↑ HR in three of four smoking cues - ↑ Attention to cues
**ALCOHOL**				
Lee et al. (2008)	−14 TS (abstinent 3 weeks), substantial AD^b^, ^c^ - Control: 14 NTS, social drinkers	- C: complex +/– social pressure. CC: VR neutral +/– social pressure - Randomized exposure	- VAS	- ↑ Craving in alcoholic group vs. social drinkers in complex environment - ↑ Craving after social pressure craving in all groups and environments except for alcoholic group in complex environment
Bordnick et al. (2008)	−40 NTS, AD^d^	- C: 4 complex (2 with social pressure) / CC: VR neutral - Randomized, 3 min exposure to each scene	- VAS - AAS	- ↑ Craving - ↑ Attention to cues - ↓ Craving and Thought about Drinking in 1 complex environment (“argument” room)
Ryan et al. (2010)	−15 NTS, binge drinkers - Control: 8, non-binge drinkers	- C: 4 complex (2 with social pressure) / CC: VR neutral - 3 min exposure to each scene	- VAS - AAS	- ↑ Craving in binge vs. non-binge drinkers in two complex environments (1 with social pressure) - ↑ Attention to cues in both groups - ↑ In Though About Drinking score in the two environments with social pressure in binge drinkers group
Traylor et al. (2011)	−14 NTS, moderate ND^a^, ^b^ / intermediate AD^b^, ^c^ - Control: 7 NTS, moderate ND^a^, ^b^/daily drinkers	- C: 2 complex smoking cues / CC: VR neutral - 3 min exposure	- VAS	- ↑ OH craving in OH group vs. non-OH group in one complex environment - ↑ Nicotine craving in both groups.
Kim and Lee (2015)	−18, HSD^e^ - Control: 18, LSD^f^	- C: 4 complex / CC: 4 VR neutral - Randomized exposure	- VAAT (Duration of push or pull response) - AUDIT, BDI	- ↑ Attention to cue in HSD - ↑ Depression score in HSD vs. LSD
**COCAINE**				
Saladin et al. (2006)	−11 NTS, CoD^b^	- C: 7 contextual or complex /CC: VR neutral - Randomized, 3 min exposure	- VAS - HR, SCR	- ↑ Craving - ↑ HR in four cocaine-related environments - No difference in SCR
**CANNABIS**				
Bordnick et al. (2009)	−20 NTS, CaD^b^	- C: 1 proximal and 1 complex / CC: VR neutral - Randomized, 6 min exposure	- VAS - CAS	- ↑ Craving - ↑ Attention to cues
**GAMBLING DISORDER**				
Bouchard et al. (2017)	−28 NTS, frequent gamblers^g^ - Control: 36 NTS, occasional gamblers^g^	- C: 2 complex / CC: real VLT and real board game - Randomized, 7 min exposure	- GCS - SOGS	- ↑ Craving (anticipation and desire scores) vs. real board game in frequent gamblers group - Craving correlated with dependence
**INTERNET GAMING DISORDER**				
Shin et al. (2018)	-−34 NTS, IGD^h^, ^i^ - Control: 30, healthy subjects (adolescents and young adults)	- C: 4 complex - Randomized exposure	- VAS - Modified-IAT - PQ, SSQ	- ↑ Craving in IGD group vs. control group - Craving correlated with dependence in one environment in IGD group - ↑ SSQ in IGD group vs. control group

a *FTND*.

b *DSM-IV*.

c *ADS (Alcohol dependence scale)*.

d *DSM-IV-TR*.

e *AUDIT > 8*.

f *AUDIT < 8*.

g *SOGS*.

h *DSM-V*.

i *occasional or frequent problems because of internet at IAT*.

*AAS, Alcohol Attention Scale; ACVAS, Attention to Cue Visual Analog Scale; AD, Alcohol dependent; AUDIT, Alcohol Use Disorder Identification Test; BDI, Beck Depression Inventory; C, Cue; CaD, Cannabis dependent; CAS, Cannabis, Attention Scale; CC, Control Cue; CoD, Cocaine dependent; CT, Controlled Trial; fMRI, functional Magnetic Resonance Imaging; FTND, Fagerström Test for Nicotine Dependence; GCS, Gambling Craving Scale; HR, Heart Rate; HSD, Heavy, Social Drinkers; IAT, Young Internet Addiction Test; IG, Internet Gaming; LSD, Light Social Drinkers; MDS, Multi-Dimensional Scale; ND, Nicotine dependent; NTS, Non Treatment Seeking; OH, alcohol; PFC, Pre-Frontal Cortex; PQ, Presence Questionnaire; QSU, Questionnaire of Smoking Urge; RCT, Randomized Controlled Trial; SAS, Smoking Attention Scale; SCR, Skin Conductance Response; SOGS, South Oak Gambling Scale; SoSQ, attitude toward Sense of Smell Questionnaire; SR, Skin Resistance; T, Temperature; TS, Treatment Seeking; VAAT, Virtual Approach Avoidance Task; VAS, Visual Analog Scale; VLT, Video Lottery Terminal; VR, Virtual Reality*.

#### Nicotine

Seventeen studies (*n* = 465) assessed cue reactivity after virtual nicotine-related cues in adult smokers. Nicotine addiction criteria were reported in 13 studies, using DSM 4 (Guze, 1995) and/or Fagerström Test for Nicotine Dependence criteria (Heatherton et al., 1991). Dependence severity was disparate across 8 studies, ranging from low to high (Lee et al., 2003, 2005; Traylor et al., 2008, 2009, 2011; Acker and MacKillop, 2013; Gamito et al., 2014; Thompson-Lake et al., 2015). Virtual reality exposure was controlled by a randomized healthy control group in 2 studies (Traylor et al., 2011; Gamito et al., 2014) and active 2D image comparators in 2 others (Lee et al., 2003, 2005). In most of the remaining studies, virtual nicotine cues were controlled by virtual neutral environments with random exposure protocols. Virtual environments included multisensory exposure with visual, auditory and olfactory stimuli in 8 studies (Carter et al., 2008; Traylor et al., 2008, 2009, 2011; Kaganoff et al., 2012; Acker and MacKillop, 2013; García-Rodríguez et al., 2013; Thompson-Lake et al., 2015) and visual and auditory environment for others (Ferrer-García et al., 2010; Paris et al., 2011; Pericot-Valverde et al., 2011; Acker and MacKillop, 2013; García-Rodríguez et al., 2013; Thompson-Lake et al., 2015).

##### Craving

All studies investigating craving (*n* = 445) in VR showed craving induction in adult smokers. Various cues (proximal: lighter, ashtray, pack of cigarettes, contextual: convenience store, party, complexes: smokers at a party or at a pub, having lunch at home) were associated with craving induction.

In most cases (17–24, 26, 27, 29, 32, 33), complex cues were evaluated and often compared to other types of cues (Bordnick et al., 2005; Traylor et al., 2008; Paris et al., 2011; Pericot-Valverde et al., 2011; Kaganoff et al., 2012; Gamito et al., 2014; Thompson-Lake et al., 2015). Some results suggested greater craving induction following complex cues compared to proximal (Thompson-Lake et al., 2015) or contextual cues (Gamito et al., 2014). Pericot-Valverde et al. (2011) suggested that social pressure in the form of virtual avatar could induce a faster craving increase in a complex-cue environment, although no difference in overall craving intensity was found. Conversely, García-Rodríguez et al. (2013) showed that smoking a virtual cigarette could act as a stronger proximal cue on craving induction compared to complex situations (García-Rodríguez et al., 2013). Altogether, 3 out of 7 studies failed to find differences in craving induction between types of cues and there are several possible explanations for these results. First of all, the limited use of random exposure protocol could have exposed craving measurements to a report effect, which therefore could limit cues comparability; only one study truly controlled this bias (Thompson-Lake et al., 2015). Secondly, most studies performed craving assessment using single-item visual analog scales, reducing craving to a single dimension, and being then more sensitive to the ceiling effect. Conversely, multidimensional scales can highlight interesting craving differences by assessing it on various motivational dimensions using the Questionnaire of Smoking Urges (QSU) (Thompson-Lake et al., 2015) or through a neuro-economic assessment of craving as proposed by Acker and MacKillop (2013) which found subjects more likely to spend money on cigarettes, continue to smoke even at higher cost and are less sensitive to cigarette prices. Finally, Traylor et al. showed no difference in nicotine craving between subjects who are nicotine and alcohol dependent vs. nicotine deprendent only subjects after exposure to virtual complex environments (Traylor et al., 2011). The relationship between craving induction and dependence levels in VR were mixed and understudied with only one positive result showing predictive value of dependence and withdrawal scores on craving intensity (Thompson-Lake et al., 2015), while no correlation between craving and cigarette consumption was found in Ferrer-García et al. (2010), this possibly reflects a lower nicotine dependence in the smoker group. Surprisingly, no studies evaluated the effect of sensorial diversity on craving, which was supposed to increase immersion, especially since presence and craving levels were correlated in one study (Ferrer-García et al., 2010).

##### Physiological response

The physiological response to virtual nicotine-related cues was assessed in 5 studies (*n* = 145 participants) (Bordnick et al., 2005; Lee et al., 2005; García-Rodríguez et al., 2012, 2013; Thompson-Lake et al., 2015). Results are inconsistant. On the one hand, proximal and complex cues triggered increased skin conductance (Bordnick et al., 2005) and heart rate (García-Rodríguez et al., 2013; Thompson-Lake et al., 2015) compared to neutral environments in smokers. On the other hand, complex cues were associated with decreased heart rate and no change in skin resistance in smokers compared to non-smokers (García-Rodríguez et al., 2012). Ultimately, these contradictory findings may reflect a low nicotine dependence in the smoker group. Temperature was assessed in one study only (García-Rodríguez et al., 2012) and they found that its unfrequent increase in only 2 out of the 7 environments. Finally, one functional MRI study showed a pre-frontal cortex activation in response to the VR nicotine stimuli compared to neutral stimuli (Lee et al., 2005).

##### Attention to cue

3 studies evaluated the impact of virtual nicotine-related cues on attentional bias by using the self-administered questionnaires (ACVAS) or the Smoking Attention Scale (SAS) (Traylor et al., 2009; Kaganoff et al., 2012; Thompson-Lake et al., 2015) (*n* = 102). All studies reported a significant increase of attentional bias compared to virtual neutral environments. Adding olfactory nicotine cues were surprisingly not associated with greater attention to cue in Traylor et al. (2009) study. One study showed the feasibility of using implicit measurement of attentional bias in VR through eye-tracking and found greater ocular fixations on nicotine cues in smokers compared to non-smokers (Gamito et al., 2014).

#### Alcohol

Five studies (*n* = 101) assessed cue reactivity after presentation of virtual alcohol-related cues in adults suffering from alcohol use disorder (Bordnick et al., 2008; Lee et al., 2008; Ryan et al., 2010; Traylor et al., 2011; Kim and Lee, 2015). Diagnosis criteria were reported in 4 studies (Bordnick et al., 2008; Lee et al., 2008; Traylor et al., 2011; Kim and Lee, 2015) using DSM 4 (Guze, 1995) or Alcohol Use Disorder Identification Scale [AUDIT (Saunders et al., 1993)]. Dependence level was disparate and ranged from intermediate to severe dependence [AUDIT or Alcohol Dependence Scale (ADS) (Kivlahan et al., 1989)] in 3 studies (Lee et al., 2008; Traylor et al., 2011; Kim and Lee, 2015). Virtual alcohol-related cues were controlled with virtual neutral environment in each study and randomly exposed in 3 studies. Four studies included a control group (Lee et al., 2008; Ryan et al., 2010; Traylor et al., 2011; Kim and Lee, 2015) of which one was randomized (Traylor et al., 2011). Virtual environments included a multisensory exposure with visual, auditory and olfactory stimuli in 3 studies (Bordnick et al., 2008; Ryan et al., 2010; Traylor et al., 2011) and visual and auditory stimuli in 2 studies (Lee et al., 2008; Kim and Lee, 2015).

##### Craving

All studies investigating craving (*n* = 93) showed its induction after VR cue exposure (Bordnick et al., 2008; Lee et al., 2008; Ryan et al., 2010; Traylor et al., 2011). Cues usually associated with alcohol consumption (proximal: bottle of alcohol, contextual: bar, complex: party with alcohol) were those inducing craving. All studies investigated craving through single-item VAS. Three studies (Bordnick et al., 2008; Lee et al., 2008; Ryan et al., 2010) studied social interaction impact on craving. Bordnick et al. (2008) found that social pressure (such as offering a cigarette in a party) in addition to complex cues showed no difference in craving level whereas negative social experiences were associated to craving reduction in subjects with alcohol use disorders. Lee et al. (2008) found similar results regarding social pressure in alcohol dependent subjects; unlike with social drinkers for which craving was increased, suggesting a greater sensitivity to the social context in this population (Ryan et al., 2010). Finally, Traylor et al. assessed cross-cue reactivity and didn't find differences in alcohol craving after nicotine-related cue exposure among nicotine and alcohol dependent subjects compared to nicotine only dependent subjects (Traylor et al., 2011). These results may however have been limited by the duration of alcohol abstinence (12 h) which could have result in a ceiling effect, reflected by a greater baseline craving.

##### Attention to cue

Two studies assessed attentional bias upon virtual alcohol-related cue exposure using the self-report questionnaire Alcohol Attention Scale (AAS) (Bordnick et al., 2008; Ryan et al., 2010). Attentional bias increased every virtual complex cue compared to virtual neutral environments among alcohol dependent subjects (Bordnick et al., 2008). Ryan et al. (2010) found that the subscore “Thinking about alcohol” was higher after social pressure for the binge drinker group compared to healthy controls. Through implicit measurement (with a Virtual Approach-Avoidance Task), Kim and Lee (2015) highlighted attentional bias in heavy social drinkers (HSD) that showed increased difficulty avoiding virtual alcohol-related situations compared to light social drinkers. High depression score may however have affected response time in the HSD group, thus limiting this result.

#### Cocaine

One study (Saladin et al., 2006) concerning adults (*n* = 11) suffering from cocaine use disorder (DSM-IV) (Guze, 1995) assessed cue reactivity after virtual cocaine-related cues compared to virtual neutral environment, randomly exposed. Virtual environments included visual and auditory cues.

##### Craving

Craving induction through virtual cues has been demonstrated with contextual and complex environments compared to neutral virtual environments in cocaine-dependent subjects. Craving assessment was performed using single-item VAS. Situations triggering highest craving are those related to cocaine use and interactions with a dealer.

##### Physiological response

Physiological reactivity was also measured. Heart rate increased in a manner comparable to craving in scenes of cocaine use and interaction with a dealer compared to neutral scenes. Besides, there was no change in skin conductance measurements between virtual neutral exposure or virtual cocaine-related exposure.

#### Cannabis

One study (Bordnick et al., 2009) concerning adults (*n* = 20) suffering from cannabis use disorder (DSM-IV) (Guze, 1995) assessed cue reactivity after virtual cannabis-related cues were introduced. The study was controlled with a virtual neutral environment that was randomly exposed. Virtual environments consisted of multisensory exposure with visual, auditory and olfactory cues.

##### Craving

Bordnick et al. (2009) showed craving induction after virtual proximal and complex cues compared to virtual neutral stimuli in cannabis dependent subjects. Craving assessment was performed using single-item VAS.

##### Attention to cue

In this study (Bordnick et al., 2009), sub-scores of the 3 items of the Cannabis Attention Scale (CAS) self-questionnaire was increased after cannabis cues in VR compared to neutral stimuli in VR.

#### Gambling Disorder

One study (Bouchard et al., 2017) assessed cue reactivity after virtual gambling-related cues in 28 male frequent gamblers [SOGS (Lesieur and Blume, 1987)]. Virtual gambling cue exposure was controlled with 1 real complex environment (video lottery terminal) and 1 real neutral environment (board game), randomly exposed. Virtual environments consisted of multisensory exposure with visual and auditory cues.

##### Craving

Craving (both anticipation and desire to play dimensions) were induced after exposure to a virtual and real slot machine environments in Bouchard et al. (2017). Craving assessment was performed using multi-dimensional scale CGS. Increase in desire was higher compared to “casual gamers.” Moreover, craving measured by the total score of the GCS was significantly correlated to gambling dependence.

#### Internet Gaming Disorder

One study (Shin et al., 2018) concerning adolescents and young adults (*n* = 34) suffering from internet gaming disorder [DSM-5 (Petry and O'Brien, 2013)] controlled with a group of 30 healthy subjects assessed cue reactivity after virtual internet gaming-related cues. Virtual environments consisted of multisensory exposure with visual and auditory cues.

##### Craving

Shin et al. (2018) found induced craving after randomized exposure to 4 virtual complex internet gaming-related cues (cyber-cafes). Craving assessment was performed using a single-item VAS. Craving was higher in the 2 active environments (coffee entrance and gaming invitation task) compared to a passive observation of a gaming conversation. In the environment associated with the task of refusal skills practice, the use of coping with gaming invitation strategies reduced craving compared to other environments in the IGD group. In addition, craving level and severity of dependence (modified-Internet Addiction Test) were correlated in the “coffee entrance” situation within the IGD group and a trend was found in the observation and gaming invitation situation. Although cybersickness score was higher in the IGD group compared to the control group, scores reported in both groups were low by SSQ (Simulator Sickness Questionnaire) standards.

#### Treatment

Studies and results described below are summarized in Table 3.

**Table 3:** Clinical trial on Virtual Reality (VR) treatment in addiction.

**Study**	**Design**	**Population**	**Control conditions**	**VR intervention**	**A**	**Assessment**	**Outcomes**
**NICOTINE**							
Lee et al. (2004)	- Trial	−15 adolescent males, low to moderate ND^1^		- VET (20 min, 1 session) 20 min sessions	N	- Baseline, end of treatment: VAS, morning and daily smoking count, planning (min), FTND, SSQ, PQ	- No change in craving and others variables
Pericot-Valverde et al. (2014)	- Trial	−48 TS, low to moderate ND^a^, ^b^		- VET: progressive individualized exposure (30 min, 5 sessions, 1/w)	Y	- Baseline, end of treatment: VAS, cig/d, air expired CO	- ↓ Craving - ↓ Cig/day and air expired CO
Pericot-Valverde et al. (2015)	- Trial	−41 TS, low to moderate ND^a^, ^b^		- VET: degressive individualized exposure (30 min, 5 sessions, 1/w)	N	- Baseline: gender, age, years of education, marital status, duration of daily smoking, FTND, NDSS, MNWS, STAI, BDI-II, DD - Week 1 and 5 post-treatment: VAS	- ↓ Craving; correlated with younger age, higher Cig/day, DD, BDI-II
Park et al. (2014)	- CT	−30 TS males, moderate ND^a^, ^c^	- TTT: CBT (4 sessions, 1/w)	- VET: 2 complex and 2 neutral (25 min, 4 sessions, 1/w)	N	- Baseline, end of treatment, week 12: QSU, cig/d, air expired CO, FTND, MNWS (per protocole analysis)	- No change in craving- No difference on craving - ↓ Dependence (daily smoking count, expiratory CO levels, FTND)
Pericot-Valverde et al. (2019)	- RCT	−102 TS, moderate ND^a^, ^c^	- TTT: CBT (60 min, 6 sessions, 1/w)	- VET (+CBT): progressive individualized exposure (30 min, 5 sessions, 1/w)	N	- Baseline and month 1, 6, 12: VAS, abstinence, relapse rate, treatment retention (ITT analysis)	- ↓ Craving - ↑ Relapse at 12 months
Girard et al. (2009)	- RCT	−91 outpatients, moderate to high ND^a^	- TTT: Grasp up to 60 virtual balls (30 min, 4 sessions, 1/w)	- VBT: find and crush up to 60 virtual cigarettes (30 min, 4 sessions, 1/w)	N	- Baseline, end of treatment, week 12: cig/d, air expired CO, FTND, PQ, SSQ (ITT analysis)	- ↓ Dependence - ↑ Abstinence at week 12 - ↓ Treatment drop out - ↑ Presence and ↓ cybersickness at end of treatment
Bordnick et al. (2012)	- RCT	−46 TS, moderate to high ND^a^, ^d^	- TTT: NRT	- VCBT: progressive individualized exposure and coping skill training (1 h, 10 sessions, 1/w)	Y	- Baseline, end of treatment: QSU-Brief, cig/d, SASE - 1 month post treatment: SCQ (No ITT analysis)	- ↓ Craving - ↓ cig/d - ↑ Self-efficacy and SCQ
**ALCOHOL**							
Lee et al. (2009)	- CT	−38 inpatient males, AD^d^(abstinent from a week)	- TTT: CBT + education (45 min, 10 sessions, 2/w) - Group: VET in 15 healthy males	- VET: relaxation, exposure, aversive situation (25 min, 10 sessions, 2/w)	Y	- Baseline, end of treatment: VAS, EEG	- ↓ Craving - ↑ Right Frontal EEG alpha power
Son et al. (2015)	- Trial	−12 inpatient, AD^d^, ^e^ (abstinent from a week)	- Group: 15 healthy subjects	- VET: relaxation, exposure, aversive situation (25 min, 10 sessions, 2/w)	Y	- Baseline, end of treatment: VAS, TEP-FDG	- ↓ Craving - No correlation between change in craving and brain metabolism
Choi and Lee (2015)	- Trial	−20 male, HSD^e^	- Group: 20 male LD^f^	- VET: 2 social aversive situations (20 min, 1 session)	N	- Baseline, end of treatment: AUQ, alcohol-IAT, eye-tracking test, alcohol-Stroop test.	- ↓ Craving - ↓ Alcohol-IAT and reaction times for alcohol-related stimuli
**GAMBLING DISORDER**							
Giroux et al. (2013)	- Trial	−10 outpatient, gamblers		- VET: progressive exposure (20 min, 1 session)	Y	- Baseline, post treatment: VAS, self-efficacy	- No change in craving and self-efficacy
Bouchard et al. (2017)	- CT	- Study 1: 28, frequent gamblers^g^	- TTT: *in vivo* exposure or neutral exposure (1 session) - Group: 36 occasional gamblers^g^	- VET: 2 complex (7 min each, 1 session)	Y	- Baseline, post treatment: GCS, SOGS	- ↑ Craving in VR gambling and real VLT; correlated to baseline SOGS; correlated to baseline SOGS
		- Study 2: 34 inpatient, pathological gamblers^b^ undergoing CBT	- TTT: imaginal exposure (2 sessions)	- VET: 2 complex (20 min, 2 session)	Y	- Baseline, post treatment: GCS, SSQ	- ↓ Craving
		- Study 3: 25, pathological gamblers^b^ undergoing CBT	- TTT: imaginal exposure (4 sessions)	- VET: 2 complex scenes (20 min, 4 session)	Y	- Baseline, post treatment: My treatment, questionnaire, effectiveness (CPGI, DIG, GRCS)	- ↓ Craving - ↑ Effectiveness
**INTERNET GAMING DISORDER**							
Park et al. (2016)	- RCT	−24, IGD^h^	- TTT: CBT (2 h, 8 sessions, 2/w)	- VET: relaxation, exposure, aversive situation (2 h, 8 sessions, 2/w)	Y	- Baseline, post treatment: YIAS, fMRI	- ↓ YIAS in both conditions - ↑ Connectivity from the PCC seed to the left middle frontal and bilateral temporal after VET

a *FTND*.

b *DSM-IV-TR*.

c *DSM-V*.

d *DSM-IV*.

e *AUDIT >8*.

f *AUDIT <8*.

g *SOGS*.

h *IYAS>50*.

*A, Assisted by a therapist; AUDIT, Alcohol Use Disorder Identification Test; AUQ, Alcohol Urge Questionnaire; CBT, Cognitive-Behavioral Therapy; CO, Carbone monOxyde; CPGI, Canadian Problem Gambling Index; CT, Controlled Trial; DD, Delay Discounting; DIG, Diagnostic Interview for Gambling; EEG, Electroencephalography; FDG-PET, Fluoro-Deoxy-Glucose Positron Emission Tomography; FTND, Fagerström Test for Nicotine Dependence; GCS, Gambling Craving Scale; GRCS, Gambling Related Cognition Scale; HSD, Heavy social drinkers, IAT, Implicit Association Test; ITT, Intention To Treat; LD, Light Drinkers; MNWS, Minnesota Nicotine Withdrawal Scale; NDSS, Nicotine Dependence Syndrome Scale; NTS, Non-Treatment Seeking; PCC, Posterior Cingulate Cortex; PQ, Presence Questionnaire; QSU, Questionnaire of Smoking Urge; RCT, Randomized Controlled Trial; SASE, Smoking Abstinence self-efficacy; SCQ, Smoking Confidence Questionnaire; SSQ, Simulator Sickness Questionnaire; STAI, State-Trait Anxiety Inventory; TS, Treatment Seeking; VAS, Visual analog Scale; VBT, Virtual Behavioral Therapy; VCBT, Virtual cognitive-Behavioral Therapy; VET, Virtual Exposure Therapy; VLT, Video Lottery Terminal; YIAS, Young Internet Addiction Scale; YIAS, Young Internet Addiction Scale*.

#### Nicotine

##### Virtual Exposure Therapy (VET)

5 VET trials (Lee et al., 2004; Park et al., 2014; Pericot-Valverde et al., 2014, 2015, 2019) assessed craving and nicotine addiction reduction in nicotine dependent adults (*n* = 241). One VET trial was effective on craving and cigarette consumption reduction (controlled on exhaled CO concentration) after 5 weekly sessions on 48 subjects with low to moderate nicotine dependence in association with a tobacco education group (Pericot-Valverde et al., 2014). In a second trial, the team showed an association between craving reduction and younger age, higher cigarette consumption, impulsivity and depressive symptoms with the same exposure protocol (Pericot-Valverde et al., 2015). However, 1 CT and 1 RCT (Park et al., 2014; Pericot-Valverde et al., 2019) found mixed results (Pericot-Valverde et al., 2019). In the first study, similar changes were observed in both VET and CBT groups on cigarette consumption reduction, exhaled CO concentration and dependence score post-treatment and at follow-up in moderate dependent subjects (Park et al., 2014). However, craving decrease wasn't significant in either group and higher baseline Fagerström score in the CBT group may have limited comparability. The second study, a RCT combination of VET plus CBT for smoking cessation, showed no benefit on retention and cessation rate compared with CBT alone after randomization in 102 nicotine moderate dependent subjects (Pericot-Valverde et al., 2019). While VET was associated with a reduction in nicotine craving, the 1-year relapse rate was higher in the VET + CBT group. Explanation concerning this last surprising result could be that cue induced craving responses extinguished after CET protocol re-emerged, a phenomenon known as spontaneous recovery. Otherwise, individuals assigned to CBT only protocol may have over time developed and rehearsed strategies for coping with both craving and withdrawal symptoms, reducing their susceptibility to relapse.

##### Virtual Cognitive Behavioral Therapy (VCBT)

Two randomized controlled trials (n = 137) studied VCBT in nicotine addiction reduction (Girard et al., 2009; Bordnick et al., 2012).

The first VBT program consisted of finding and crushing 60 cigarettes in a virtual environment without therapist assistance. Implemented 4 weekly in conjunction with a psychosocial support program, the VBT was effective on nicotine addiction reduction and abstinence rate in 91 moderate to high dependent smokers after treatment and during follow-up compared to a virtual task control (Girard et al., 2009).

The second VCBT consisted of 10 weekly sessions including a gradual and personalized exposure to craving with training in coping skills guided by a therapist. Implemented in combination with a nicotine replacement therapy, the VCBT was effective on cigarette consumption and craving reduction compared to nicotine replacement therapy alone in 46 moderates to high dependent smokers (Bordnick et al., 2012). In addition, the retention rate and self-confidence at the end of the intervention and coping skills during follow-up were better in this group. However, these results should be interpreted with caution because of low overall adherence to treatment.

#### Alcohol

Three controlled studies (*n* = 70) in Korea evaluated virtual exposure therapy, including aversive exposure situations on alcohol craving reduction (Lee et al., 2009; Choi and Lee, 2015; Son et al., 2015).

VET was more effective than a standardized CBT on reducing alcohol craving after 10 biweekly sessions in 38 males who were abstinent for a week. The virtual exposure included relaxation time, craving and aversive exposure guided by a therapist (Lee et al., 2009). Using the same protocol, this team found no correlation between craving reduction and brain metabolism in FDG-PET in 12 patients abstinent for 1 week (Son et al., 2015). On the other hand, VET was effective on craving reduction in heavy social drinkers after one session compared to light consumers (Choi and Lee, 2015). The exposure consisted of two aversive social situations including visual and auditory stimuli not supervised by a therapist. Moreover, HSD's ranking of alcohol markers in positive categories decreased (alcohol-IAT behavioral task) after treatment and VET was associated with decreased ocular fixation times for alcohol-related markers and improved alcohol-Stroop test scores for both groups.

Despite these encouraging results, evidence of VET effectiveness on alcohol dependence reduction had still not been studied.

#### Gambling Disorder

One study found no reduction on urge to gamble after a single VET on 10 lottery players (Giroux et al., 2013). More recently, pilot data from Bouchard et al. showed promising results (Bouchard et al., 2017): after demonstrating the effectiveness of a VET protocol on reducing desire to gamble similarly to a real-life exposure in 28 frequent players, they found no difference in craving reduction between randomized VET and imagined exposure on 34 pathological gamblers, both group receiving standardized CBT. Finally, 4 VET sessions were associated with stronger sense of control over gambling-related disorders after the third exposure compared to an exposure in imagination after randomization on 25 pathological players.

#### Internet Gaming Disorder

One VET study showed dependence reduction after 8 biweekly sessions compared to standard CBT on 24 randomized subjects (Park et al., 2016). The VET consisted of relaxation time followed by personalized craving and aversive cue exposure assisted by a therapist, with positive reinforcement. However, no craving measure was performed.

## Discussion

In the present review, we discuss major results related to VR that may provide solutions in the field of addiction especially for assessment and treatment.

Several results suggest that immersing subjects in virtual environments related to an addiction can combat many issues regarding craving assessment (Hone-Blanchet et al., 2014; Pericot-Valverde et al., 2016). VR have proved to be effective in triggering craving in both substance use disorder and behavioral addiction. Specifically designed to be immersive, the VR multisensorial cue exposition is able to drive cue attentional bias in order to report cognitive distortion related to addiction and trigger interoceptive reaction such as heart rate variation.

Although lack of statistical power makes it difficult to expose differences in craving response between types of cues, some results from nicotine craving studies (cf table) suggest that greater craving can be triggered by complex environments. Moreover, recent interest in proximal stimuli has emerged (virtual cigarettes) and new studies on other substance consumption behavior in virtual reality could further enrich the exposition realism.

Social interaction with avatars can efficiently induce cravings for multiple substances, mainly assessed in cigarette smoking and alcohol studies. However, difficulties persist in separating social craving from context craving. Indeed, several questions remain regarding stimuli specification assessment in VR craving induction. A significant number of studies were exposed to carry over effect while a better understanding of craving stimuli characteristics could be obtained by using a two by two comparison protocols or by an effective carry over effect control (for example, by controlling a return to baseline of craving level between each active stimulus exposure).

The lack of stimuli comparability resulting from the craving ceiling effect may have been increased by VAS widespread use by limiting the measurement to its intensity level, while a dimensional analysis of scales such as QSU could have revealed interesting qualitative differences (Rosenberg, 2009). More ecological assessments (eye tracking or virtual approach-avoidance task) could be proposed in order to provide an objective craving assessment by the use of real time behavioral measures (Cox et al., 2014). Its implementation in future research would help craving assessment but also offer insight and motivation to treatment via a feedback use. Feeling of presence and cybersickness should be assessed more rigorously to improve the level of ecological validity while increasing the acceptability of VR, especially since these two components are negatively correlated (Weech et al., 2019).

Traditional Cue Exposure Therapy studies' designs are mainly on substance-related cues detached from social environmental or *in vivo* scenarios. However, metanalyses report disappointing results (Mellentin et al., 2017; Ferreri et al., 2018), highlighting methodological biases preventing clear conclusions on its effectiveness in addictive disorders. VR has the potential to exceed those limits by providing a virtual immersion in environments closely related to everyday life and typical drug administration scenarios in order to better trigger craving in an individualized and progressive level. Indeed, Virtual Exposure Therapy (VET) has shown to be effective on craving reduction in nicotine and gambling disorders. Korean studies Lee et al. (2009), Son et al. (2015), and Choi and Lee (2015) find interesting results by adding aversive expositions to VET in alcohol use disorder. The combination of positive and negative affective states could lead to a restructuring of the conditioned stimulus response relation in addiction and should eventually result in avoidance of the administered drug.

In the future, attention to potential limits should be considered:

- Cost-effective studies are needed.
- Access to VR and therapists training need to be evaluated.
- Ethical considerations remain regarding the use of aversive exposures.
- Cybersickness could represent a threat to virtual reality usability and effectiveness and would also require integration in future studies (Gallagher and Ferrè, 2018).
- Patient's adherence should be accurately assessed before implementing aversion therapy in routine practice.

Both positive and negative studies show numerous methodological biases: confusion bias due to the absence of control groups, selection bias due to the absence of randomization, attrition bias due to little intent to treat analysis, altogether indicating low internal validity and limiting the possibility of drawing clear conclusions.

Future studies appear necessary and should evaluate dependence and abstinence maintenance as primary endpoint, offer more long-term follow-up, and include more control treatment groups to limit confusion bias.

Efficacy of treatment based exclusively on virtual exposure to drug related cues appears to be limited, but interesting results point the great potential of implementing VR in CBT. Pericot-Valverde et al. (2019) shows how VR could be disruptive by pointing out how an unsupervised behavior task in VR, such as finding and crushing cigarettes, can achieve positive results by making it enjoyable. Besides, it suggests that integrating several concepts such as patient empowerment to increase self-efficacy and self-regulation with more advances models on addiction associating action cue-exposure to positive mood state could achieve a full embodied experience for addiction behavior change in VR (Riva et al., 2017).

Despite these limitations, VR has the potential to offer new opportunities for treatment through its ability to provide a more ecological environment with more control and safety over exposition. The ability to fine-tune environments composition according to patient's preferences and most relevant data makes it a future tool for personalized medicine.

Because VR has the potential to be disruptive [for review (Freeman et al., 2017)] effort should be made to ensure good acceptability among therapist by providing reliable information, developing interest and allowing them to contribute to the development of these new technologies (Bourla et al., 2018). Furthermore, there is an urgent need to specifically assess the user experience. Virtual reality tends to develop in psychiatric practice (Freeman et al., 2017). Although its acceptability seems good in patients with mental disorders (Navarro-Haro et al., 2017), it has yet to be evaluated among health professionals. Its diffusion can challenge our professional heritage, the younger generations being potentially more sensitized to it. Efforts should be made through greater theoretical and practical information, especially since its application for health professionals could allow them to improve their skills in the assessment and treatment of addictive disorders (Fleming et al., 2009).

Besides its acceptability, its accessibility also raises questions. It could be limited due to the lack of financial support. The populations suffering from substance disorders being frequently in situation of social precariousness. The question of the cost of VR will also have to be evaluated in order to consider its wide implementation in health centers.

## Conclusion

The studies presented in this review suggest that VR provide benefits in the assessment and treatment of substance use disorders, behavior addictions and achieve high levels of ecological validity. While, craving provocation in VR is effective across addiction disorders, treatments based exclusively on virtual exposure to drug related cues as shown heterogenous result. The addition of learning coping strategies in VRCBT studies are promising, however more rigorous methodological studies are warranted.

## Data Availability Statement

The datasets generated for this study are available on request to the corresponding author.

## Author Contributions

TS and TB screening and abstract analysis, both authors contributed equally to the writing. AB, SM, and FF conceptual framework, supervised development of work, helped in data interpretation and manuscript evaluation. C-SP and J-VB helped to evaluate and edit the manuscript.

### Conflict of Interest

The authors declare that the research was conducted in the absence of any commercial or financial relationships that could be construed as a potential conflict of interest.
